# Real world outcome analysis of treosulfan-based conditioning prior to allo-HCT in patients with MDS compared to clinical trial data

**DOI:** 10.1038/s41409-024-02456-3

**Published:** 2024-11-05

**Authors:** Matthias Stelljes, Katja Sockel, Matthias Floeth, Johannes Schetelig, Martin Bornhäuser, Christian Reicherts, Georg Lenz, Thomas Schroeder, Miroslaw Markiewicz, Hélène Labussiere-Wallet, Péter Reményi, Fabio Ciceri, Imran Khan, Uwe Pichlmeier, Xieran Li, Friedrich Stölzel

**Affiliations:** 1 Department of Medicine/Hematology and Oncology, University Hospital of Muenster, Muenster, Germany; 2 Department of Internal Medicine, University Hospital Carl Gustav Carus, TU Dresden, Dresden, Germany; 3 Department of Bone Marrow Transplantation, West German Cancer Centre, University of Duisburg-Essen, Essen, Germany; 4 Department of Haematology, Institute of Medical Sciences, College of Medical Sciences, University of Rzeszow, Rzeszów, Poland; 5 Department of Haematology, Centre Hospitalier Lyon-Sud, Hospices Civils de Lyon, Lyon, France; 6 St István and St László Hospital of Budapest, Budapest, Hungary; 7 Haematology and Bone Marrow Transplantation Unit, Istituto di Ricovero e Cura a Carattere Scientifico, San Raffaele Scientific Institute, Milan, Italy; 8 medac GmbH, Wedel, Germany; 9 Division of Stem Cell Transplantation and Cellular Immunotherapies, Department of Internal Medicine II, University Hospital Schleswig-Holstein, Kiel, Germany

**Keywords:** Drug development, Clinical trials

## To the Editor:

Allogeneic hematopoietic cell transplantation (alloHCT) is the only curative therapy for myelodysplastic syndrome (MDS), particularly in patients with higher risk MDS, resulting in superior survival compared to non-transplant approaches in transplant eligible patients [1, 2].

Various studies demonstrated that treosulfan in combination with fludarabine showed a favorable acute toxicity profile, allowing donor cell engraftment with complete and sustained donor hematopoietic chimerism after alloHCT combined with good tolerability also for MDS patients [3–6]. To compare treosulfan- versus busulfan-based conditioning in elderly and/or comorbid patients, we performed an analysis on the MDS subgroup of a randomized phase 3 trial, which evaluated both conditioning regimens in patients with acute myeloid leukemia and MDS. In addition, to demonstrate transition of the clinical efficacy of treosulfan-based conditioning from the clinical trial setting to the real-world, the trial data was combined with data obtained from 2 large transplant centers (real-world data [RWD]).

## Subjects and methods

### Randomized controlled trial

MC-FludT.14/L was a randomized (1:1), parallel-group, open-label, multicenter, international, group-sequential phase 3 non-inferiority trial [3]. Patients aged between 50 and 70 years or with haemopoietic cell transplantation—comorbidity index (HCT-CI) > 2 in younger adult patients ≥18 years with acute myeloid leukemia or MDS (with blast counts < 20% in bone marrow during disease history) were enrolled in this trial. Here, the subgroup of patients with MDS (n = 199) was extracted from the clinical trial data set.

### Real-world data

Patients included in this analysis were treated with treosulfan-based conditioning before alloHCT (August 2017 to February 2023) at the University Hospitals Muenster and Dresden who also participated in the RCT. These data were recorded locally and provided for comparison between RCT and RWD.

Conditioning therapy was identical to the RCT, consisting of 150 mg/m^2^ fludarabine and 30 g/m^2^ treosulfan. Most patients received graft-versus-host disease (GvHD) prophylaxis with cyclosporin A and a short course of methotrexate (n = 99). In addition, anti-T-cell globulin (ATG Neovii) was given in 69 patients transplanted from unrelated donors.

### Statistical analysis plan

Descriptive statistics was applied to summarize efficacy and safety endpoints for comparison of RCT and RWD. All time-to-event endpoints were measured from the day of alloHCT (except chronic GvHD from Day +100) to the time of event or competing event and analyzed by appropriate methods indicated in the footnote of Table 1.

**Table 1:** Relapse-free survival, secondary outcomes, and GvHD (all MDS patients).

	RCT (N = 84)	RWD (N = 111)	Sensitivity analysis—PSM	
			RCT (N = 47)	RWD (N = 47)
**Relapse-free survival**				
Patients without event, n (%)	54 (64.3%)	78 (70.3%)	29 (61.7%)	35 (74.5%)
Relapse-free survival at 24 months, % (95% CI)^a^	68.1 (56.6, 77.2)	66.8 (55.5, 75.8)	68.7 (52.5, 80.4)	65.5 (45.2, 79.8)
Hazard ratio (RWD/RCT) (95% CI)^b^	0.85 (0.52, 1.39)		0.73 (0.35, 1.52)	
p value^c^	0.5138		0.3945	
**OS**				
Patients without event, n (%)	57 (67.9%)	83 (74.8%)	32 (68.1%)	39 (83.0%)
Overall survival at 24 months, % (95% CI)^a^	72.8 (61.5, 81.3)	72.4 (61.6, 80.6)	74.8 (58.6, 85.4)	76.7 (57.7, 88.0)
Hazard ratio (RWD/RCT) (95% CI)^b^	0.80 (0.47, 1.36)		0.56 (0.24, 1.33)	
p value^c^	0.4168		0.1853	
**Relapse**				
Patients with event	11 (13.1%)	15 (13.5%)	9 (19.1%)	5 (10.6%)
Cumulative incidence at 24 months, % (95% CI)	10.8 (4.1, 17.5)	14.9 (7.2, 22.6)	14.9 (4.7, 25.1)	14.1 (1.1, 27.2)
Hazard Ratio (RWD/RCT) (95% CI)^d^	1.12 (0.51, 2.43)		0.63 (0.21, 1.85)	
p value^e^	0.7567		0.4151	
**NRM, n (%)**				
Patients with event	19 (22.6%)	18 (16.2%)	9 (19.1%)	7 (14.9%)
Cumulative incidence at 24 months, % (95% CI)	21.1 (12.0, 30.1)	18.3 (10.2, 26.4)	16.4 (4.9, 27.9)	20.3 (6.2, 34.5)
Hazard Ratio (RWD/RCT) (95% CI)^d^	0.73 (0.38, 1.39)		0.87 (0.32, 2.34)	
p value^e^	0.3472		0.7803	
**Engraftment**				
***Neutrophil, n (%)***				
Cumulative incidence at 28 days, % (95% CI)	92.9 (87.3, 98.4)	93.6 (89.1, 98.2)	95.7 (90.0, 100.0)	91.5 (83.5, 99.5)
Duration of neutrophil engraftment, days Median (Min, Max)	19.0 (11, 42)	15.0 (11, 42)	18.0 (12, 25)	17.0 (11, 42)
Hazard Ratio (RWD/RCT) (95% CI)^d^	1.36 (1.05, 1.77)		1.13 (0.76, 1.67)	
p value^e^	0.0513		0.6170	
***Platelet, n (%)***				
Cumulative incidence at 28 days, % (95% CI)	89.3 (82.7, 95.9)	92.7 (87.8, 97.6)	91.5 (83.5, 99.5)	93.5 (86.3, 100.0)
Duration of platelet engraftment, days Median (Min, Max)	14.0 (8, 38)	14.0 (6. 79)	14.0 (8, 38)	14.0 (9, 43)
Hazard Ratio (RWD/RCT) (95% CI)^d^	1.03 (0.79, 1.35)		1.03 (0.70, 1.52)	
p value^e^	0.8199		0.8688	
**Acute GvHD**				
Patients with any acute GvHD, n [%]	46 (54.8%)	44 (39.6%)	24 (51.1%)	19 (40.4%)
Odds ratio (95% CI)	0.54 (0.31, 0.96)		0.65 (0.29, 1.47)	
p value^f^	0.0359		0.3006	
**Chronic GvHD**				
Patients with any chronic GvHD, n [%]	44 (52.4%)	46 (41.4%)	22 (46.8%)	23 (48.9%)
Odds ratio (95% CI)	0.64 (0.36, 1.14)		1.08 (0.48, 2.45)	
p value^f^	0.1292		0.8364	

*CI* confidence interval, *GvHD* graft-versus-host disease, *Max* maximum, *MDS* myelodysplastic syndrome, *Min* minimum, *N* total number of patients, *NRM* non-relapse mortality, *PSM* propensity score matching, *RCT* randomized controlled trial, *RWD* real-world data, *OS* overall survival.

^a^Based on Kaplan-Meier estimates.

^b^Cox regression model (unadjusted).

^c^Log-rank test.

^d^Fine and Gray model (unadjusted).

^e^Test of Gray.

^f^Crude Chi-Square test.

A sensitivity analysis was conducted using propensity score matching (PSM) with a 1:1 ratio through nearest neighbor without replacement and a caliper of 0.1. Patients were matched on the score from a logistic regression on predefined prognostic factors and all additional baseline variables that were significantly (p < 0.1) different between the RCT and RWD.

## Results

### Comparison between RCT and RWD

#### Baseline characteristics

195 MDS patients (84 RCT, 111 RWD) received treosulfan prior to alloHCT and were included in this comparison. Median age was 62 years (range: 39–76); patients in the RWD group were older. No significant difference of disease characteristics between the RCT and RWD groups was observed except more higher risk patients, more patients with neutrophil count ≥0.8 g/L, and higher HCI-CI scores. Blast count in bone marrow was well-balanced between groups (Supplementary Table 1).

#### Efficacy

Overall, the median (range) follow-up time was 26.9 (3.0, 51.2) months and 24.1 (3.2, 55.6) months in the RCT and RWD groups. Kaplan-Meier estimate of relapse-free survival at 24 months was comparable between RCT and RWD groups (68.1% versus 66.8%; p = 0.5138) (Table 1, Supplementary Fig. 1).

In this analysis, overall survival was 72.8% (95% confidence interval [CI]: 61.5, 81.3) in the RCT and 72.4% (95% CI: 61.6, 80.6) in the RWD group (Table 1). The Kaplan-Meier estimates showed no difference (Supplementary Fig. 1).

At 24 months, 10.8% (95% CI: 4.1, 17.5) patients in the RCT and 14.9% (95% CI: 7.2, 22.6) patients in the RWD group relapsed and 21.1% (95% CI: 12.0, 30.1) of patients in the RCT and 18.3% (95% CI: 10.2, 26.4) of patients in the RWD group died without relapse (Table 1).

Neutrophil engraftment at Day +28 after HCT was achieved by 92.9% (95% CI: 87.3, 98.4) of RCT patients and 93.6% (95% CI: 89.1, 98.2) of RWD patients (p = 0.0513). The median duration of neutrophil engraftment was longer in the RCT (19.0 days versus 15.0 days) (Table 1).

The sensitivity analysis was repeated in patients selected through PSM. In addition to the predefined factors, baseline neutrophil was included for the propensity score derivation due to imbalance. Balance for all baseline characteristics after matching was demonstrated (Supplementary Table 1) and the overall standardized mean difference was 0.05. The results showed a comparable efficacy profile of RWD compared to RCT (Table 1 and Supplementary Fig. 2).

#### Safety

At the time of this comparative analysis, 32.1% of patients in the RCT group and 25.2% of patients in the RWD group had died. Transplantation-related causes were the leading cause of death (20.2% in RCT, 14.4% in RWD) followed by relapse/progression (4.8% in RCT, 9.0% in RWD). The incidence of acute GvHD (Grade I-IV) was 39.6% in the RWD and 54.8% in the RCT. In total, 41.4% patients in the RWD experienced chronic GvHD compared to 52.4% patients in the RCT (Table 1).

## Discussion

This analysis confirms results from recently published randomized trials, demonstrating the substantial curative potential of alloHCT with treosulfan-based conditioning, even in elderly and/or comorbid MDS patients. In the RCT, compared to busulfan-based reduced intensity conditioning, the treosulfan-fludarabine regimen showed a trend towards better survival outcomes with significantly improved event-free survival at 24 months post-HCT (Supplementary Fig. 3). This advantage was mainly due to improvement of toxicity with lower non-relapse mortality, demonstrating a major clinical benefit for the new treosulfan-fludarabine regimen [3, 6].

Treosulfan before alloHCT in real-world setting resulted in comparable efficacy when compared with results from the RCT. The survival rates in the present comparison of more than 70% about 2 years after transplantation seem to be very promising because they were observed robustly in both the clinical trial and the real-world settings as well as in other clinical studies [5, 7, 8]. Of note, disease burden prior HCT, as a classical prognostic factors for MDS patients, defined as percentage of bone marrow blast (e.g. <10% versus 10–20%) did not impact patients’ survival significantly (data not shown).

The primary limitation of this analysis is the timing of RWD collection, which was conducted after RCT. RWD data was obtained from University Hospitals Muenster and Dresden after they completed participation in the RCT and hence, there is no treatment time overlap. This timing discrepancy with potentially improved standard of care and concomitant treatment may have contributed to, numerically, slightly better relapse-free survival and overall survival in the RWD set compared to RCT data. Furthermore, baseline and disease characteristics between the two data sets revealed some heterogeneities. Therefore, we also conducted a PSM which only allowed for a small pre-specified amount of difference between the propensity scores of RWD and RCT patients.

In conclusion, the benefit of the new treosulfan regimen over a busulfan regimen was consistently observed for relapse-free survival, overall survival, and non-relapse mortality, and could be translated into real-world environment, which suggests that this regimen has the potential to become a standard preparative regimen before alloHCT in patients with MDS not eligible for standard myeloablative conditioning.

## Supplementary information

Supplemental data
