# Supplementary material for: Real world outcome analysis of treosulfan-based conditioning prior to allo-HCT in patients with MDS compared to clinical trial data
Source: Bone Marrow Transplant. 2024 Nov 5;60(1):103–6. doi: 10.1038/s41409-024-02456-3 (PMC11726458; doi:10.1038/s41409-024-02456-3)
Supplement: Supplementary file 1 — Supplemental data [file 41409_2024_2456_MOESM1_ESM.docx]

SUPPLEMENTAL DATA

(Stelljes et al. Correspondence)

This supplemental data contains supporting supplemental tables and figures that are referred in the correspondence.

Table of Contents

[Table of Contents 2](#_Toc178691213)

[List of Supplemental Tables 2](#_Toc178691214)

[List of Supplemental Figures 2](#_Toc178691215)

[Supplemental Information 3](#_Toc178691216)

List of Supplemental Tables

[Supplemental Table 1: Baseline Demographics and Disease Characteristics (All MDS Patients) 5](#_Toc178691219)

List of Supplemental Figures

[Supplemental Figure 1: MDS Subgroup Analysis in MC-FludT.14/L 7](#_Toc178691222)

[Supplemental Figure 2: Kaplan-Meier Estimates of Relapse-free Survival and Overall Survival (All MDS Patients) 8](#_Toc178691223)

[Supplemental Figure 3: Kaplan-Meier Estimates of Relapse-free Survival and Overall Survival (Propensity Score Matched MDS Patients) 9](#_Toc178691224)

Supplemental Information

*Randomized Controlled Trial*

The primary objective was to compare event-free survival (EFS) within 2 years after allogeneic haematopoietic stem cell transplantation (alloHCT) between treosulfan- and busulfan-based conditioning regimens. Events were defined as relapse/progression, graft failure, or death (whichever occurred first). A total of 570 patients were enrolled of whom 551 received treosulfan (268) or busulfan (283) and underwent alloHCT.

*Real-world Data*

Patients were allografted with peripheral blood stem cells from matched related (n=17), 10/10 HLA-matched unrelated (n=75), 9/10 HLA-matched unrelated (n=16) or haploidentical donors (n=3).

A combination of tacrolimus and mycophenolate mofetil (MMF) was given in 2 patients, 1 patient received cyclosporin A and MMF, 1 patient received cyclosporin A only, and 8 patients received post-transplant cyclophosphamide combined with a calcineurin inhibitor and MMF.

*Statistical Analysis Plan*

The probability of event over time for EFS (only for comparison of treosulfan and busulfan in randomized controlled trial (RCT), defined as time to events of relapse of disease, graft failure, or death), relapse-free survival (RFS, defined as time to relapse or death) and overall survival (OS, defined as time to death) was estimated by Kaplan-Meier methods. The probability of event over time with competing risks for non-relapse mortality (NRM) and relapse was estimated by cumulative incidence functions. Cox proportional hazards models for RFS and OS, and Fine and Gray models for NRM and relapse were applied to adjust statistical analysis for covariates in multivariate analyses. Probabilities of acute graft-versus-host disease (GvHD) and chronic GvHD were compared by similar time-to-event analysis for comparison of treosulfan and busulfan in RCT, whereas they are compared by odds ratio with Chi-square test between RCT and real-world data due to data availability. Subgroup analyses were conducted focusing on baseline morphologic blast count and the Revised International Prognostic Scoring System (IPSS-R). All analyses were performed with SAS software (Version 9.4) and R (Version 4.2.2).

Supplemental Table 1: Baseline Demographics and Disease Characteristics (All MDS Patients)

|  | **RCT** | | **RWD** | | **Sensitivity Analysis - PSM** | |
| --- | --- | --- | --- | --- | --- | --- |
|  | **(N=84)** | | **(N=111)** | | **RCT** | **RWD** |
|  |  | |  | | **(N=47)** | **(N=47)** |
| **Sex, n (%)** |  | |  | |  |  |
| Female | 26 (31.0%) | | 39 (35.1%) | | 13 (27.7%) | 18 (38.3%) |
| Male | 58 (69.0%) | | 72 (64.9%) | | 34 (72.3%) | 29 (61.7%) |
| *P* (Chi-square) | 0.5395 | | | | 0.2727 | |
| **Age, years; mean (SD)** | 59.2 (6.4) | | 62.7 (6.5) | | 60.5 (5.8) | 60.7 (7.1) |
| *P* (ANOVA) | 0.0003 | | | | 0.8740 | |
| **Blasts, n (%)** |  | |  | |  |  |
| Blasts <5% | 38 (45.2%) | | 46 (41.4%) | | 18 (38.3%) | 17 (36.2%) |
| Blasts 5-10% | 33 (39.3%) | | 44 (39.6%) | | 20 (42.6%) | 23 (48.9%) |
| Blasts >10% | 12 (14.3%) | | 21 (18.9%) | | 9 (19.1%) | 7 (14.9%) |
| Missing | 1 (1.2%) | | 0 (0.0%) | | 0 (0.0%) | 0 (0.0%) |
| *P* (Wilcoxon) | 0.4296 | | | | 0.9378 | |
| **MDS risk group stratification based on IPSS-R, n (%)** |  | |  | |  |  |
| Very low risk | 5 (6.0%) | | 1 (0.9%) | | 2 (4.3%) | 1 (2.1%) |
| Low risk | 15 (17.9%) | | 5 (4.5%) | | 6 (12.8%) | 4 (8.5%) |
| Intermediate risk | 18 (21.4%) | | 21 (18.9%) | | 8 (17.0%) | 10 (21.3%) |
| High risk | 22 (26.2%) | | 33 (29.7%) | | 14 (29.8%) | 18 (38.3%) |
| Very high risk | 24 (28.6%) | | 51 (45.9%) | | 17 (36.2%) | 14 (29.8%) |
| *P* (Wilcoxon) | 0.0005 | | | | 0.9370 | |
| **HCT-CI total score** |  | |  | |  |  |
| Median (Q1, Q3) | 3.0 (1.0, 5.0) | | 2.0 (0.0, 3.0) | | 3.0 (1.0, 4.0) | 2.0 (0.0, 4.0) |
| Min, Max | 0, 10 | | 0, 7 | | 0, 8 | 0, 7 |
| Missing | 0 | | 29 | | 0 | 14 |
| *P* (Wilcoxon) | 0.0029 | | | | 0.1124 | |
| **Neutrophils [G/L] [n (%)]** |  | |  | |  |  |
| <0.8 | 38 (45.2%) | | 17 (15.3%) | | 14 (29.8%) | 15 (31.9%) |
| >=0.8 | 46 (54.8%) | | 92 (82.9%) | | 33 (70.2%) | 32 (68.1%) |
| Missing | 0 (0.0%) | | 2 (1.8%) | | 0 (0.0%) | 0 (0.0%) |
| *P* (Chi-square) | <0.0001 | | | | 0.8233 | |
| **Platelets [G/L] [n (%)]** |  | |  | |  |  |
| <50 | 31 (36.9%) | | 42 (37.8%) | | 16 (34.0%) | 16 (34.0%) |
| 50-<100 | 24 (28.6%) | | 31 (27.9%) | | 15 (31.9%) | 10 (21.3%) |
| >=100 | 29 (34.5%) | | 37 (33.3%) | | 16 (34.0%) | 21 (44.7%) |
| Missing | 0 (0.0%) | | 1 (0.9%) | | 0 (0.0%) | 0 (0.0%) |
| *P* (Chi-square) | 0.9830 | | | | 0.1047 | |
| **Donor type, n (%)** |  | |  | |  |  |
| Haplo | 0 (0.0%) | | 3 (2.7%) | | 0 (0.0%) | 0 (0.0%) |
| MRD | 17 (20.2%) | | 20 (18.0%) | | 7 (14.9%) | 6 (12.8%) |
| MUD | 67 (79.8%) | 91 (82.0%) | | 40 (85.1%) | | 41 (87.2%) |
| *P* (Chi-square) | 0.2272 | | | 0.7651 | | |
| **Sex mismatch, n (%)** |  |  | |  | |  |
| Donor female / Recipient male | 12 (14.3%) | 15 (13.5%) | | 6 (12.8%) | | 9 (19.1%) |
| Other combinations | 72 (85.7%) | 96 (86.5%) | | 41 (87.2%) | | 38 (80.9%) |
| *P* (Chi-square) | 0.8771 | | | 0.3981 | | |
| **CMV (recipient/donor), n (%)** |  |  | |  | |  |
| R-/D- | 30 (35.7%) | 40 (36.0%) | | 19 (40.4%) | | 20 (42.6%) |
| R-/D+ | 13 (15.5%) | 9 (8.1%) | | 6 (12.8%) | | 3 (6.4%) |
| R+/D- | 13 (15.5%) | 21 (18.9%) | | 7 (14.9%) | | 8 (17.0%) |
| R+/D+ | 28 (33.3%) | 41 (36.9%) | | 15 (31.9%) | | 16 (34.0%) |
| *P* (Chi-square) | 0.4230 | | | 0.7711 | | |
| **Source of stem cells, n (%)** |  |  | |  | |  |
| Bone marrow | 1 (1.2%) | 0 (0.0%) | | 1 (2.1%) | | 0 (0.0%) |
| Peripheral blood | 83 (98.8%) | 111 (100.0%) | | 46 (97.9%) | | 47 (100.0%) |
| *P* (Chi-square) | 0.2491 | | | 0.3147 | | |
| Note: Missing values are excluded from p-value calculations.  ANOVA, analysis of variance; CMV, cytomegalovirus; HCT-CI, hematopoietic cell transplantation comorbidity index; IPSS-R, Revised International Prognostic Scoring System; MDS, myelodysplastic syndrome; MRD, matched related donor; MUD, matched unrelated donor; PSM, propensity score matching; RCT, randomized controlled trial; RWD, real-world data; SD, standard deviation. | | | | | | |

Supplemental Figure 1: Kaplan-Meier Estimates of Relapse-free Survival and Overall Survival (All MDS Patients)


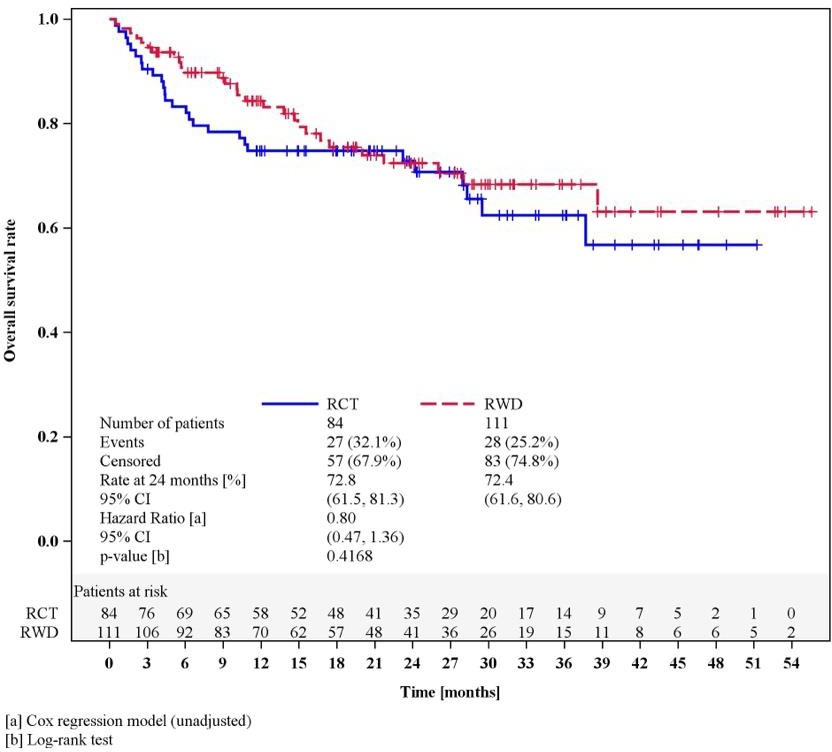


Overall Survival

B


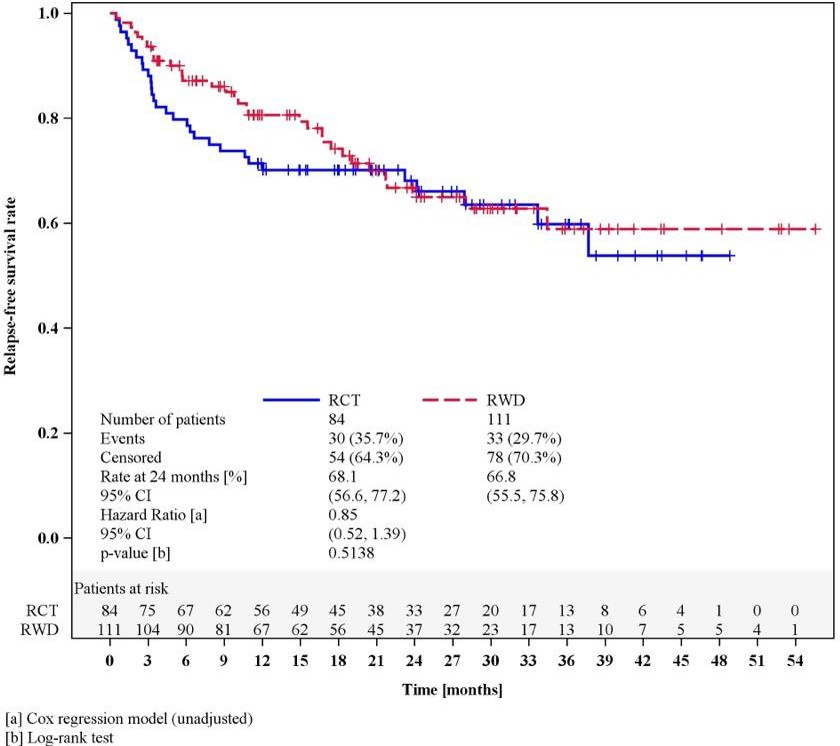


Relapse-free Survival

A

Supplemental Figure 2: Kaplan-Meier Estimates of Relapse-free Survival and Overall Survival (Propensity Score Matched MDS Patients)


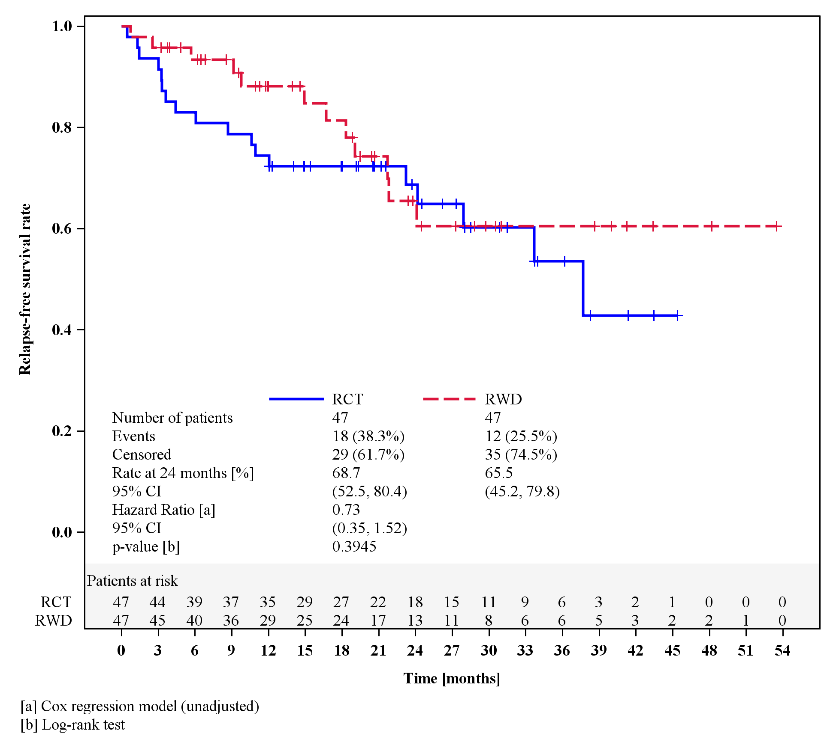

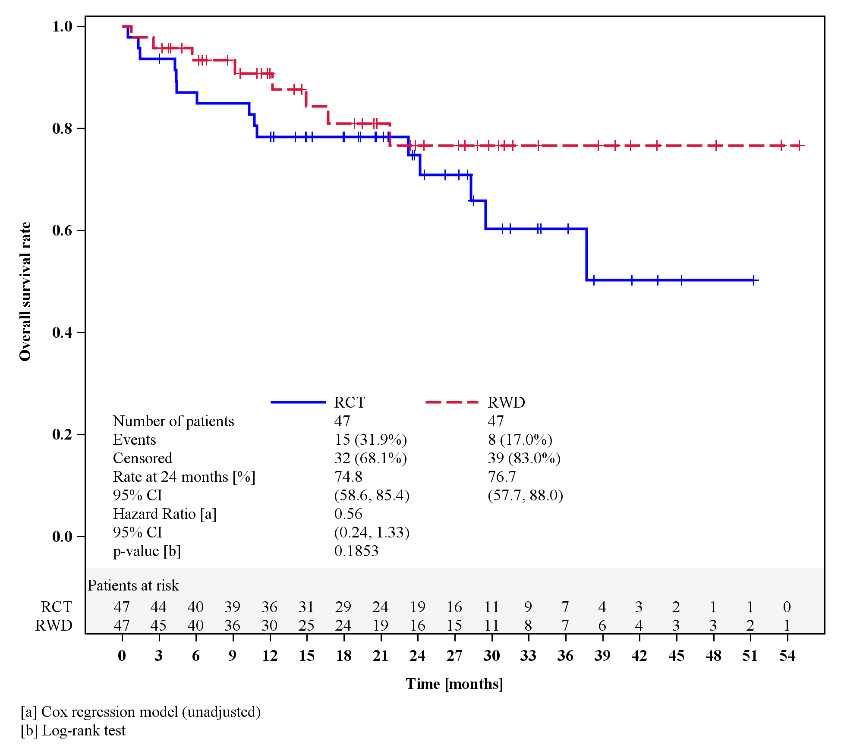


B

A

Overall Survival

Relapse-free Survival

Supplemental Figure 3: MDS Subgroup Analysis in MC-FludT.14/L


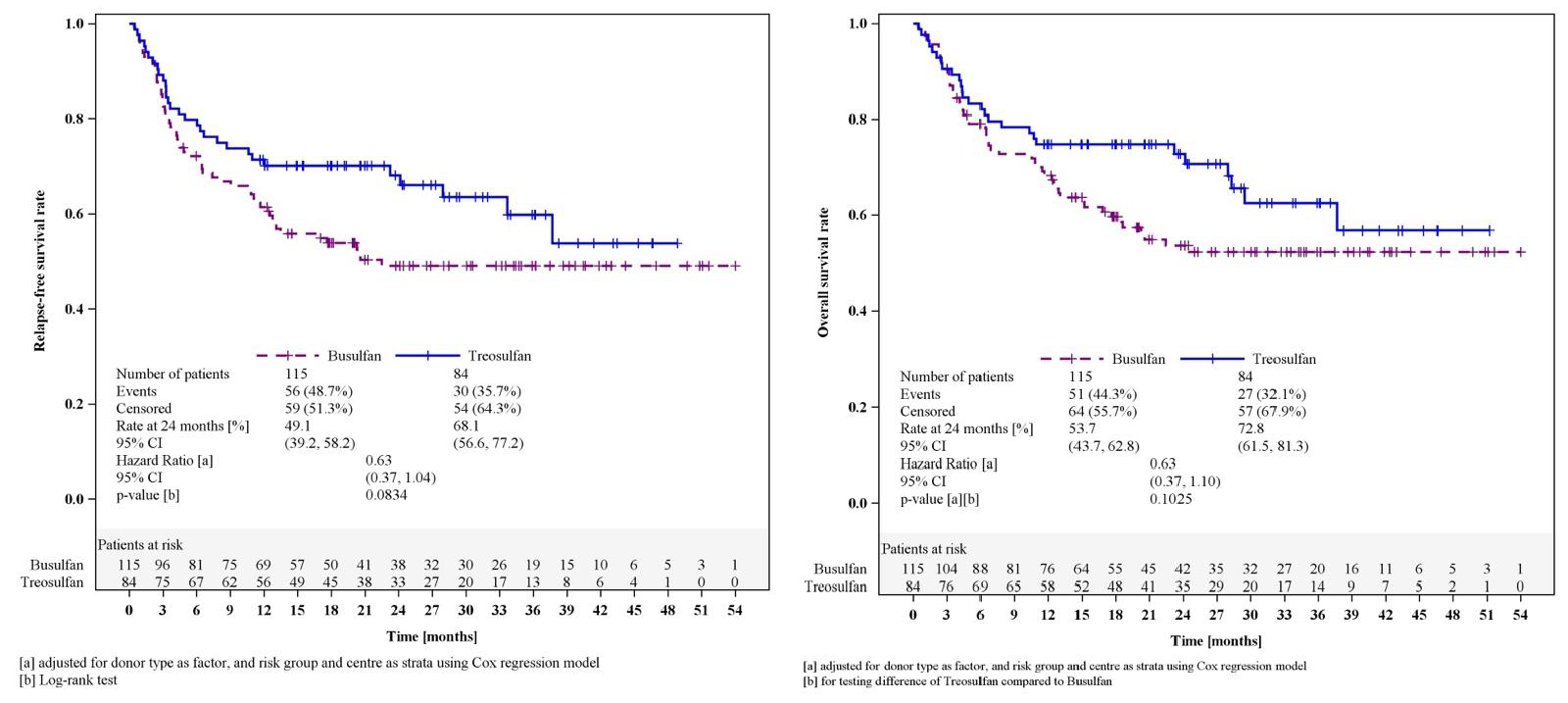


Relapse-free Survival

A

Overall Survival

B


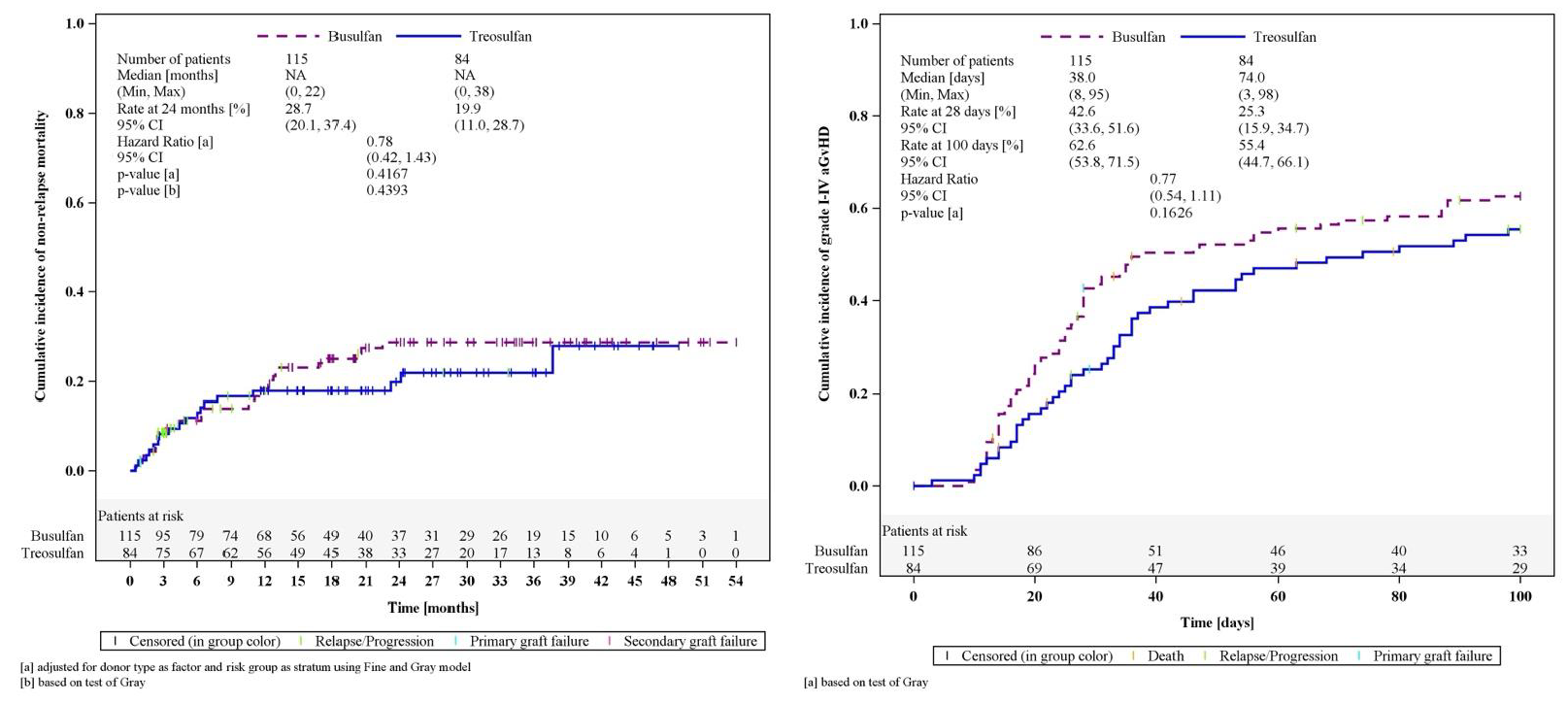


C

D

CI NRM

CI Acute GvHD


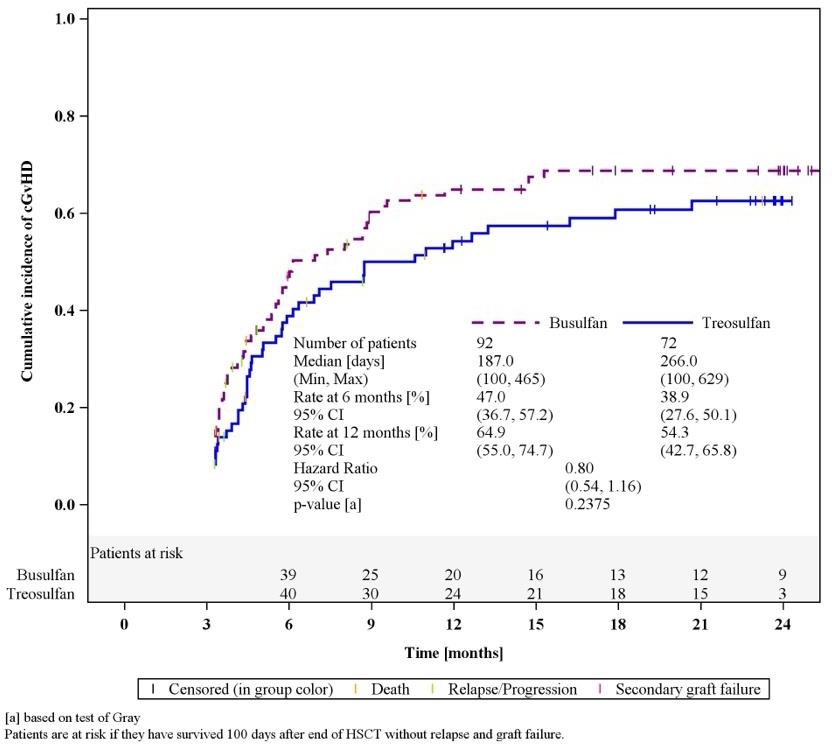


CI Chronic GvHD

E
